# Synthetic Methodologies to Gold Nanoshells: An Overview

**DOI:** 10.3390/molecules23112851

**Published:** 2018-11-02

**Authors:** Yu-Chen Wang, Éric Rhéaume, Frédéric Lesage, Ashok Kakkar

**Affiliations:** 1 Department of Chemistry, McGill University, 801 Sherbrooke St. West, Montreal, QC H3A 0B8, Canada; yu-chen.wang@mail.mcgill.ca; 2 Research Center, Montreal Heart Institute, 5000 Belanger Street, Montreal, QC H1T 1C8, Canada; 3 Department of Electrical Engineering Ecole Polytechnique de Montreal, C.P. 6079 succ. Centre-ville, Montreal, QC H3C 3A7, Canada

**Keywords:** gold nanoshells, gold nanoshell synthesis, cored gold nanoshells, hollow gold nanoshells, template galvanic replacement method

## Abstract

Gold nanostructures that can be synthetically articulated to adapt diverse morphologies, offer a versatile platform and tunable properties for applications in a variety of areas, including biomedicine and diagnostics. Among several conformational architectures, gold nanoshells provide a highly advantageous combination of properties that can be fine-tuned in designing single or multi-purpose nanomaterials, especially for applications in biology. One of the important parameters for evaluating the efficacy of gold nano-architectures is their reproducible synthesis and surface functionalization with desired moieties. A variety of methods now exist that allow fabrication and chemical manipulation of their structure and resulting properties. This review article provides an overview and a discussion of synthetic methodologies to a diverse range of gold nanoshells, and a brief summary of surface functionalization and characterization methods employed to evaluate their overall composition.

## 1. Gold Nanoshells: Introduction

**Figure 1:**
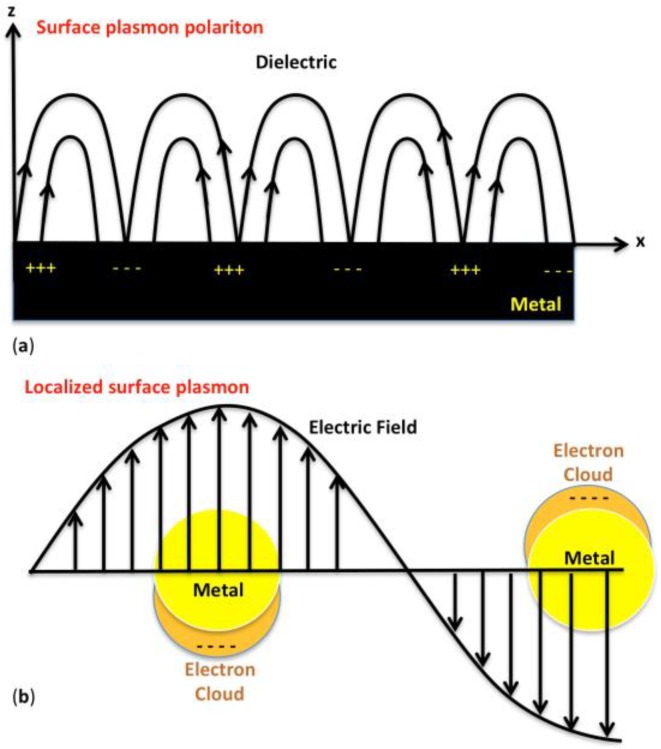
(**a**) Surface plasmon polariton; and (**b**) localized surface plasmon.

For times unknown, church windows and palaces have been bestowed with glittering colored glasses. Although the technology to impart these beautiful colors to structural materials has been known for centuries, the origin of color had remained a mystery until 1857, when Michael Faraday reduced chloroauric acid to dispersive gold nanoparticle solutions of ruby-red and pink color [1]. The theory to explain as to how nanosize metal particles possess different colors from the bulk metal wasn’t made available until about 1908. Mie explained this phenomenon using Maxwell’s equations [2], and it is now commonly referred to as surface plasmon resonance (SPR) or localized SPR. The latter is a well-known phenomenon that has a wide range of applications in areas, including non-linear optics to biosensors. The plasmon, a charged density oscillation, is the quantum energy associated with the resonant frequency of a plasma oscillation. In metals, conduction electrons (plasma) move freely, and can be excited by an electromagnetic wave, such as an optical beam. The oscillation of conduction electrons and that of the electromagnetic field are coupled in a plasmon wave, and it forms a topical area of research commonly referred to as plasmonics. Surface plasmon resonance is the collective oscillation of free conduction electrons in metals, and in other words, it relates to a propagating plasmon wave (Figure 1a). When the metal dimension is as small as a few to hundred nanometers, and the particle size is much smaller than the wavelength of the incident light as well as the penetration depth of the field, it is referred to as localized surface plasmon resonance (Figure 1b) [3,4]. In localized surface plasmon resonance, electrons are polarized by the electric field, and as a consequence it leads to accumulation of surface charge on opposite sides (Figure 1b). In a nanoparticle, this collective oscillation creates a large electromagnetic field at the surface. The plasmon resonance frequency strongly depends on the composition, size and shape of metal nanoparticles, as well as the dielectric constant of the surrounding medium [5,6,7,8,9,10,11]. Furthermore, when such particles are put in close proximity, their localized surface plasmons can interact with each other, which causes a change in resonance frequency, resulting in nanoplasmonics.

**Figure 2:**
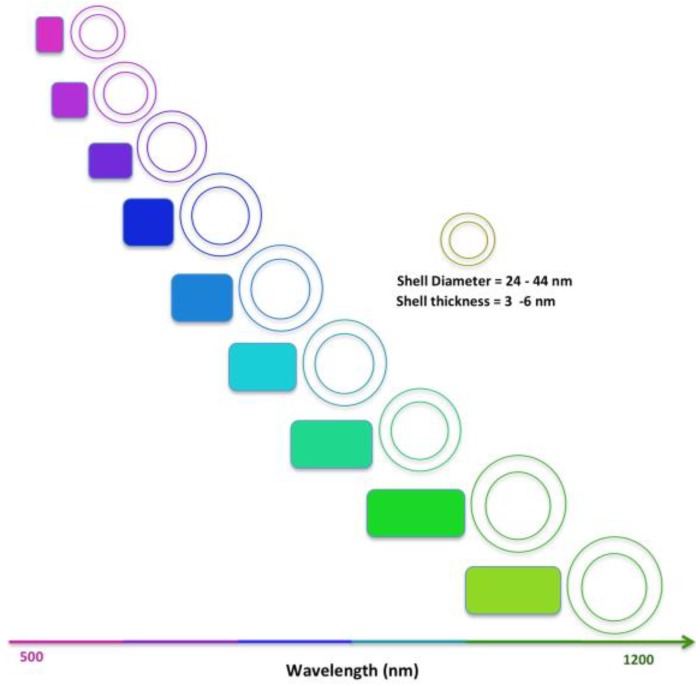
Absorption spectral range of gold nanoshells with varied shell diameter and thickness. Adapted from the data presented in reference [28].

Gold metal nanoparticles, including nanoshells, have drawn a lot of attention in the last few years because of their unique properties, including localized surface plasmon resonance. A gold nanoshell represents a spherical layer of gold around a filled or hollow core. What makes gold nanoshells particularly interesting is that their plasmon resonance can easily be tuned from visible to near-infrared (NIR) range of the electromagnetic spectrum. This region of the spectrum is also called the therapeutic window where biological tissue and fluids absorb/scatter light to the least, and enable several applications, including disease diagnosis, photothermal therapy, etc. It makes gold nanoshells good candidates for applications including photoacoustic imaging [12,13,14,15,16,17,18], photothermal therapy [19,20,21,22], and optical coherence tomography (OCT) [23,24,25,26]. Imaging demonstrations with gold nanoshells using photoacoustic imaging of living mouse brains vasculature has shown a resolution to be as small as ~100 μm [18]. PEGylated hollow gold nanoshells (PEG-HAuNS) are promising contrast agents for photoacoustic tomography (PAT) with enhanced sensitivity. In addition to size and shape, surface plasmon resonance of gold nanoshells also depends on the nanoparticle surface modification and the refractive index of the surrounding medium. Tunability in gold nanoshells makes them good optical probes for chemical or biological binding [27]. The plasmon resonance frequency of gold nanoshells is related to and can be controlled by changing the aspect ratio of shell thickness to the overall diameter (Figure 2) [28]. Although several different metal nanoshells have been reported, gold nanoshells are most common when it comes to applications in biology. This is due to the inert nature of gold, and it has also been demonstrated that they do not cause acute toxicity in liver, spleen or kidney in mice [18].

## 2. Gold Nanoshells with a Core or Hollow: Synthesis

The synthesis of gold nanoshells with an inner Au_2_S core was first reported in 1994 [29]. Subsequently, Halas’s group developed an approach to synthesize gold shell nanoparticles with a silica core, and it opened the door for applications in diagnostics [30]. Synthetic method involved gold seeds binding to a silica core through amine groups, before mixing with the growth solution that contained gold salt. It was followed by the addition of a reducing agent to grow a complete shell. By controlling the ratio of gold shell thickness to the overall diameter, the optical properties of these gold nanoparticles can be fine-tuned for a desired application. In the beginning, cores were used as templates to grow gold shells on their surfaces and obtain materials with tailored optical properties. Several different types of cores have now been incorporated to introduce multi-functional capabilities to gold nanoshells including magnetism, fluorescence and Raman sensitivity. We describe here synthetic methodologies to gold nanoshells with or without different types of cores (solid or hollow). For the solid cores, silica will be used as an example to introduce different synthetic approaches, which are generally applicable to several other solid cores.

### 2.1. Gold Nanoshells on SiO_2_ Core

#### (i) Surfactant Assisted Seeding Method

**Figure 3:**
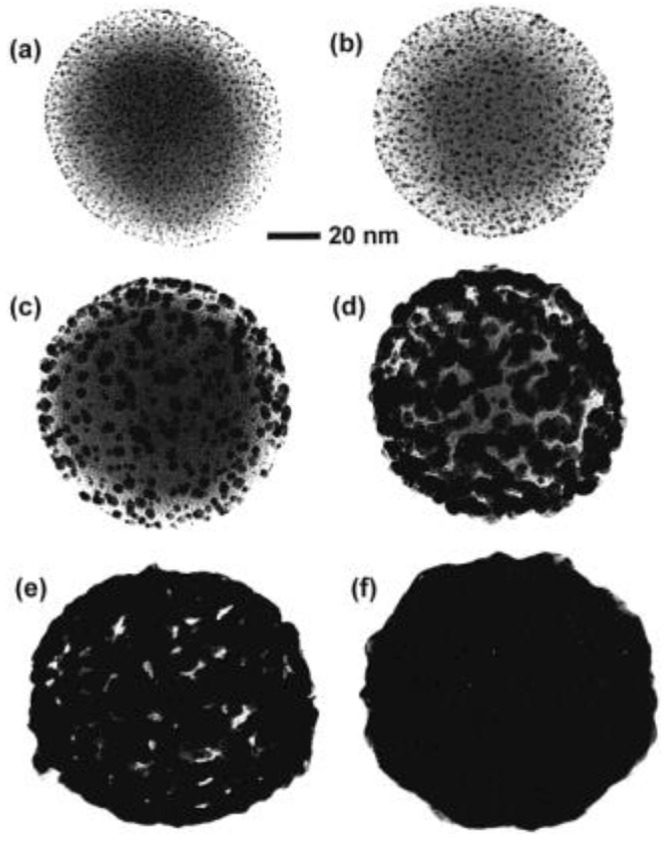
Growth of gold nanoshell depicted through transmission electron microscopy (TEM) images on a silica core: (**a**) Silica core decorated with gold colloid; (**b**–**e**) step-wise growth and coalescence of gold colloid leading to metallic gold surface layer (**f**). Reprinted with permission from Reference [30]. Copyright (1998) Elsevier.

Surfactant assisted seeding method is commonly used for the synthesis of core-shell gold nanoparticles. In a typical procedure, silica nanoparticles are first synthesized, and 3-aminopropyltriethoxysilane (APTES) as linker is condensed on their surface. It provides desired NH_2_ groups that could link to gold [30,31]. After isolating organosilane functionalized silica nanoparticles, gold colloidal solution is added to the mixture. In the first few studies by Halas’s group [30,31], it was found that about 30% surface of silica nanoparticles was covered by gold colloid, and the process was limited by inter-particle coulombic repulsions. After gold nanoparticle seeds are bound to silica nanoparticle surface, excess seeds are removed. There are two general ways to grow the gold shell on this seeded surface: (i) Using aged mixture of chloroauric acid and potassium carbonate [30], and reducing gold on nucleation sites by formaldehyde [32,33,34]; and (ii) reducing chloroauric acid with sodium borohydride [30]. Figure 3 below shows this step-wise growth of gold nanoshells using transmission electron microscopy, on a 120 nm diameter silica core [30].

A variety of capping agents on gold seeds have been used, which provide control on bonding between gold seeds and amine functionalized silica core. For example, mercaptoundecanoic acid (MUA) functionalized gold, leads to covalent linking of the seeds, through an amidation reaction between carboxylic acid end on gold seeds and amine group on the core [34,35]. Capping agent tetrakis(hydroxymethyl)phosphonium chloride (THPC) [36] can reduce repulsive force between carboxylic acid groups on gold seeds, and has been used to achieve higher coverage [37]. As compared to 3-aminopropyltrimethoxysilane (APTMS) and APTES, polymer linkers, such as polydiallyldimethyl ammonium chloride (PDADMAC) and polyethyleneimine (PEI), were found to give better coverage in both nucleation and gold nanoshell growth [38,39], and PDADMAC decorated silica core tolerated a wider range of pH [38]. Due to the availability of an abundance of amine groups on PEI, it was demonstrated that it could adsorb mid-nanometer size gold seeds (13–32 nm) through electrostatic self-assembly [39].

#### (ii) Single Sep Deposition-Precipitation (DP) Seeding Method

Deposition-precipitation method has been widely used to form supported nanoparticulate gold catalysts for carbon monoxide formation at low temperatures [40,41,42,43,44]. Compared to surfactant assisted surface seeding method, DP doesn’t require the use of gold seeds and aging, which could take up to two weeks, and is thus less time consuming. DP forms gold seeds on silica nanoparticles directly. Such a procedure developed by Olivoc group [45] involved synthesis of monodispersed silica nanoparticles with a size range of 50–440 nm. Their surface is subsequently decorated with APTES as the linker. These silica nanoparticles with amine terminal groups are then seeded with Au(OH)_3_ nanoparticles. The NH_2_ grafted silica nanoparticles enabled maintaining a uniform size and an even distribution of gold seeds compared to undecorated NH_2_ silica core. To synthesize gold nanoshells, NaOH was mixed with HAuCl_4_ to increase the pH and hydrolyze HAuCl_4_, to produce a gold hydroxide solution, which resulted in mild yellowish color. Amine grafted silica nanoparticles were then added, and the mixture heated at 70 °C for 30 min. The color of solution became orange-brown, an indication of Au(OH)_3_ nanoparticles loaded onto silica nanoparticles. Simultaneously, a colorless basic gold hydroxide solution, commonly referred to as K-gold solution, was prepared by adjusting the pH of a given amount of HAuCl_4_ solution to 10.1 by adding K_2_CO_3_, diluting it with 100 mL of water, and stirring in dark overnight at room temperature. K-gold and freshly prepared 6.6 mM NaBH_4_ (10:1 volume ratio) were added to Au(OH)_3_ seeded silica nanoparticles. A color change was observed nearly immediately, as a result of reduction of gold in Au(OH)_3_ nucleation sites. Depending on the shell thickness, the color can be red, purple, or green. It was noted that heating temperature, time, as well as NH_2_ surface modification affect gold seeds formation on silica nanoparticles. Among the six gold hydroxide species, Au(OH)_3_ is the only type of gold hydroxide that precipitates on silica nanoparticle surface. Without NH_2_ modification of silica, a heterogeneous mixture of gold nanoparticle seeds are formed that differ in size and uniformity. The equation for the gold hydroxide formation reaction is shown below: Equation (1)

AuCl_4_^−^ + *x*OH^−^ → [Au(OH)_x_Cl_4−x_]^−(x+1)^ (1)

#### (iii) Sonochemical Gold Seeding Method

Calderon-Moreno’s group used ultrasound-driven synthesis to coat silica spheres with gold nanoparticles [46]. This method does not require preparation of gold seeds, and is a greener approach to synthesis. In sonochemistry, ultrasound induces chemical changes due to cavitation phenomena involving formation, growth, and implosive collapse of bubbles in a liquid [47]. A proposed mechanism for sonochemical approach for the synthesis of gold nanoparticles by Calderon-Moreno group is shown below:

H_2_O → H^•^ + ^•^OH (2)

AuCl_4_^−^ + 3H^•^ → Au^(0)^ + H^+^ + 4Cl^−^ (3)

**Figure 4:**
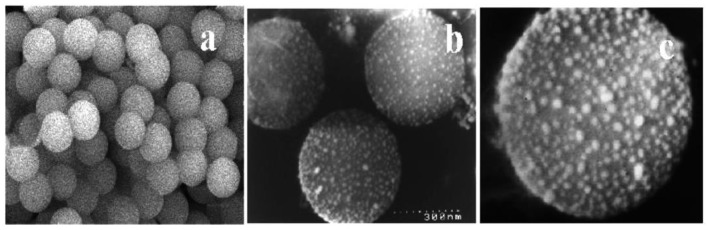
Silica submicrospheres as seen through scanning electron micrographs of (**a**) bare silica; and (**b**) coated with gold nanoparticles. The image next to (**c**) shows a higher resolution micrograph of submicrospheres coated with gold nanoparticles. Reprinted with permission from Reference [46]. Copyright (2003) American Chemical Society.

In their study, Calderon-Moreno used 200 mg of silica submicrospheres (500–800 nm in size) mixed with chloroauric acid solution, and purged the mixture with argon flow for 2 h to remove dissolved oxygen/air before sonication. 5–7 mL 24 wt % aqueous ammonia was then added during 45 min sonication radiation at 20–25 °C. Crystallization was achieved by heating at 500 °C for 3 h. Figure 4 shows scanning electron micrographs of bare silica submicrospheres, and gold nanoparticles attached to these after sonochemical procedure.

#### (iv) Sandwiched Gold Seeded Shell Synthesis

**Figure 5:**
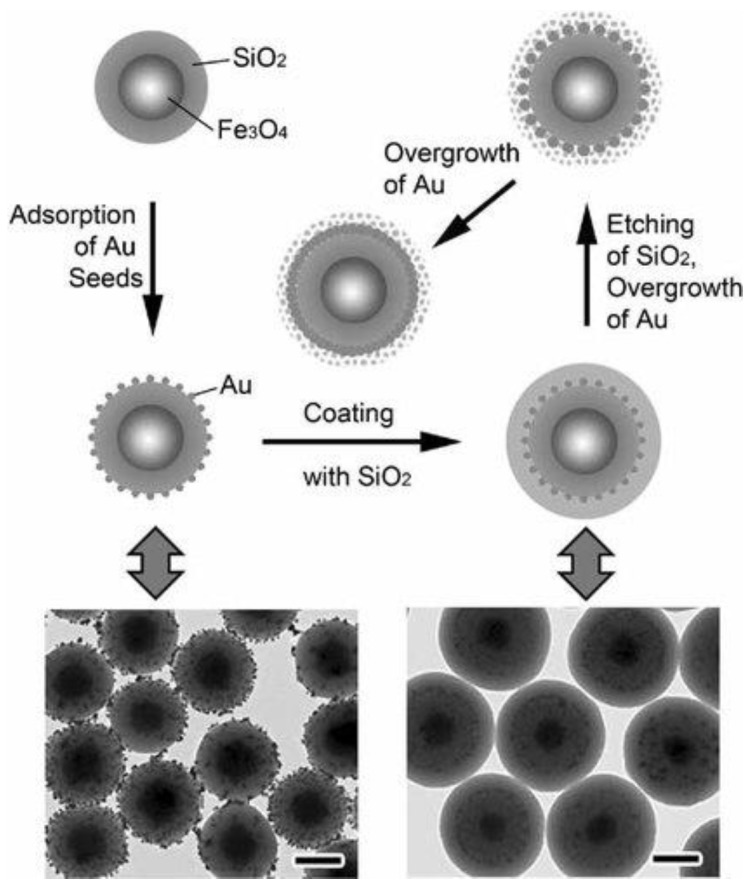
Depiction of stepwise gold nanoshell synthesis using sandwiched gold seeded approach using transmission electron microscopy. Reprinted with permission from Reference [48]. Copyright (2010) John Wiley and Sons.

To enhance reliability in controlled gold nanoshell development, a method called sandwiched gold seeded shell synthesis, has been developed, and outlined in Figure 5 [48]. In general, after gold nanoparticles have been seeded on silica nanoparticles, these are covered with another layer of SiO_2_ [49,50,51]. By using polyvinylpyrrolidone (PVP) assisted surface protected etching [52], porous silica shell is formed, which facilitates diffusion of gold seeds. Upon adding Au salt dropwise in order to have slow reaction rate and avoid self-nucleation, gold nanoshell is formed. The advantage of this method is that the confined space within a silica nanosphere can serve as a nanoreactor that favors gold particle growth anisotropically on the surface, and constrains its outward growth.

#### (v) One Pot Synthesis

As noted earlier, a general silica core based gold nanoshell synthetic strategy involves surface modification to have gold seeds attached to silica nanoparticles covalently, and subsequent sometimes weeks long process to synthesize and age gold nanoparticle seeds. Considering the time and labor intensive nature of this methodology, a process in which silica nanoparticles and gold nanoshells are sequentially formed in one pot synthesis, has been reported [53]. This method does not require functionalization of the silica surface with amines, as well as the dimensions of gold nanoshells are smaller, with a final diameter less than 30 nm and the thickness around 6 nm.

**Figure 6:**
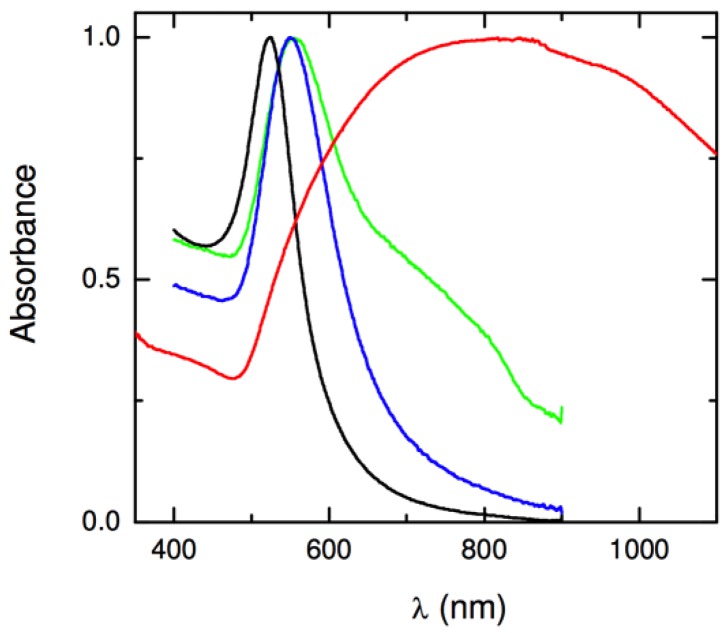
UV-Vis absorption spectral evaluation in one-pot synthesis: Black (gold nanoparticles); blue and green (incomplete nanoshells); and red: Complete gold nanoshells on silica. Reprinted with permission from Reference [53]. Copyright (2009) American Chemical Society.

The first step in one pot methodology involves hydrolysis of (3-aminopropyl)ethoxysilane by adjusting the aqueous pH to lower than 2, which leads to the formation of amino-functionalized silica nuclei [54]. After stirring for 30 min, the acidic APTES mixture is added to sodium citrate solution. With fine tuned reagent amounts and reaction time, the condensation reaction is controlled, and surface exposed amines on nanometer-size silica particles are formed. To grow gold layer on these amino-functionalized silica nanoparticles, additional sodium citrate solution is added, and the mixture is heated to 100 °C [19]. Then 20 mL of 6 × 10^−4^ M HAuCl_4_·3H_2_O solution is added slowly, in which Au^3+^ is reduced by sodium citrate, and nucleation of gold, which will coordinate with amino groups, is initiated. The solution mixture is kept at 60 °C for 30 min for complete gold layer growth, which can be confirmed by optical absorption properties, as shown in Figure 6 [53].

A similar approach was adopted by Martinez’s group, in which they used gelatin as the mediator and modify silica core surface with surface exposed amino or carboxylic groups, to bind to gold ions. It was followed by reduction of gold ions in situ to form a thin layer of gold on the silica core [55].

#### Gold Nanoshells on a Polymer Core

There are several ways to grow a thin layer of gold on polymer cores, and the most common ones are quite similar to those used for a SiO_2_ core. The general methodology is multi-step that includes (i) polymer core surface modification; (ii) attaching gold nanoparticle seeds to the surface; and (iii) then growing gold on this modified surface. The discussion below describes methods that take advantage of polymer properties, and avoid complex core surface functionalization or pre-treatment.

##### (i) Solvent Assisted Approach

**Figure 7:**
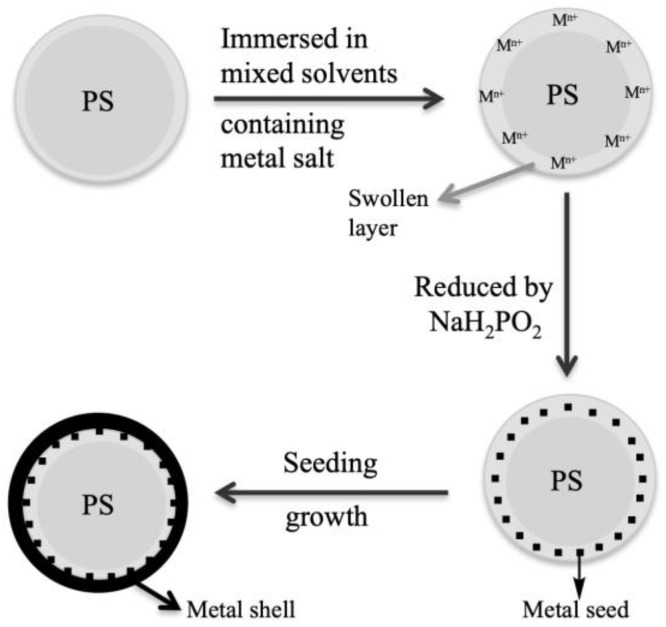
Schematics of coating polystyrene colloids with a metal shell. Redrawn by adaptation from Reference [56]. Copyright (2004) John Wiley and Sons.

Ming’s group developed a method to grow a thin layer of metal shell on a polymer core without the need of any surface functionalization for seeding [56]. They immersed polystyrene (core) in ethanol/acetone mixture that contained gold salt. The mixed solvent caused the polymer to swell, and gold ions permeated into polystyrene core. It led to the formation of a uniform layer of gold on polystyrene surface. The scheme of solvent assisted approach is shown in Figure 7 [56].

##### (ii) Combined Swelling-Heteroaggregation

**Figure 8:**
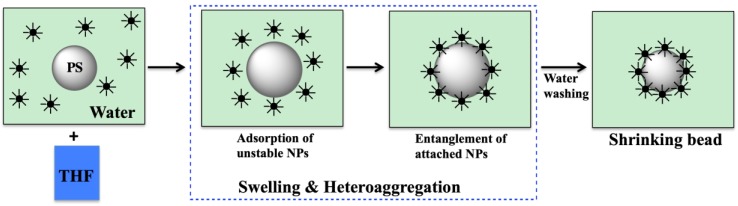
Schematic representation of a combined swelling-heteroaggregation methodology. Redrawn by adaptation from Reference [57]. Copyright (2009) American Chemical Society.

Combined swelling-heteroaggregation (CSH) is similar to solvent assisted approach, and uses a solvent to make unfunctionalized polymer core swell. But instead of using a gold precursor and reduce gold ions to gold subsequently, gold nanoparticles are directly employed [57,58]. Upon completion of the task by the polymer to form gold nanoparticles, it is dried and shrunk to trap gold nanoparticles on the surface (Figure 8) [57].

##### (iii) Gold Colloid Seeding

Seeding via gold colloids has been widely used in which a variety of methods are employed to attach colloid seeds on polymer surfaces. The gold colloid seeds either bind to the polymer via electrostatic interaction [59] or covalent bond [60]. Polystyrene is the most commonly employed core, while others, including charged polymers, have also been investigated. For example, Yang’s group [61] used positively charged polyethylenimine (PEI) to attach poly(2-methacryl(3-amide-2,4,6-triiodobenzoic acid)) (PMATIB) by electrostatic interaction to form PMATIB/PEI nanoparticles. The latter was slowly added to a gold colloid solution, which is absorbed on the surface as seeds. By mixing PAMTIB/PEI/AuNPs with sodium citrate and HAuCl_4_ aqueous solution, a thin layer of gold shell was formed that can both enhance CT contrast and photothermal therapy.

##### (iv) Gold Ion Seeding Method

**Figure 9:**
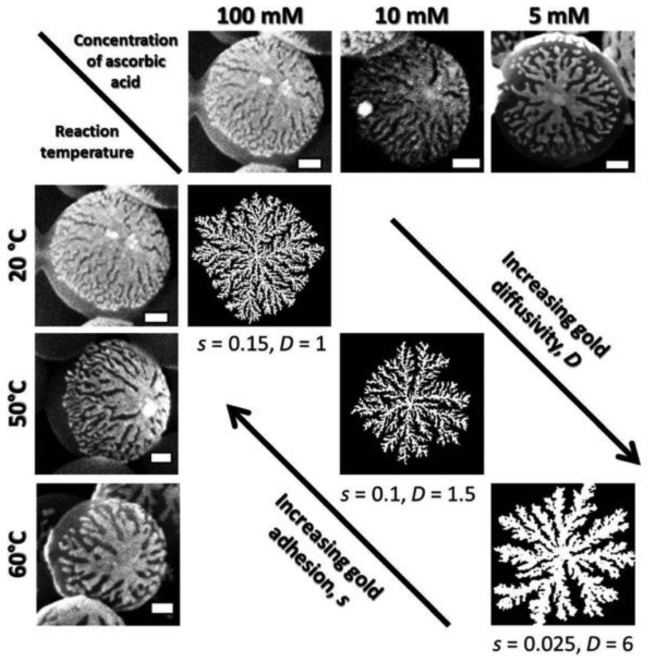
Gold ion seeding method: Effect of reaction temperature and reducing agent concentration on gold nanostructure morphology. Reprinted with permission from Reference [62]. Copyright (2014) Royal Society of Chemistry.

Klupp Taylor’s group used non-functionalized core and low metal concentration to grow an incomplete metal layer on a polystyrene core [62]. In their study, they chose polystyrene with both strong anionic and cationic character, and it avoided surface modification on the core, and gold ion could directly attach to the core surface. Reduction of gold ions was carried out in one-pot by using L-ascorbic acid. By controlling reaction conditions, such as temperature, pH and reducing agent concentration, they achieved different morphologies from dense cup-like to gold dendritic patches, which are shown in Figure 9 [62].

### 2.2. Hollow Gold Nanoshells

Synthesis of hollow gold nanoshells can be carried out by (i) using a SiO_2_ core to synthesize gold nanoshells as described above, and then using HF to remove the SiO_2_ core [63]; (ii) sacrificial galvanic replacement of cobalt nanoparticles; (iii) templated galvanic replacement of silver nanoparticles; (iv) electrochemical build-up; and (iv) employing a nonmetallic structure as core and gold as shell to prepare hollow gold nanoshells. A brief description of most commonly used methodologies to prepare hollow gold nanoshells is provided below.

#### 2.2.1. Sacrificial Template Method

Cobalt template method was first reported by Liang’s group [64], in which they followed Kobayashi method based on cobalt nanospheres as templates for synthesizing hollow platinum nanoshells [65,66]. An excess of NaBH_4(aq)_ was used to reduce CoCl_2(aq)_ in degassed water and under highly pure nitrogen flow, it led to the formation of cobalt nanospheres. The remaining unreacted NaBH_4_ was hydrolyzed that produced H_2_(g) [67]. Once H_2_ bubble formation ceased and the excess NaBH_4_ had been consumed, cobalt nanospheres solution was added to a degassed solution of HAuCl_4(aq)_. The standard reduction potentials of AuCl_4_^−^/Au and Co^2+^/Co are 0.994 and −0.277 V vs. SHE respectively. When the two solutions are mixed, electrons are transferred from cobalt to Au^3+^ ion to form Au^0^. The following are chemical equations for the two-step synthesis of hollow gold nanoshells [68,69,70]:

2NaBH_4_ + CoCl_2_ → Co^(0)^ + H_2_O + 2NaCl + B_2_H_6_ (4)

2NaBH_4_ + H_2_O → B_2_H_6_ + H_2_ + 2NaOH (5)

3Co + 2AuCl_4_^−^ → 3Co^2+^ + 2Au^(0)^ + 8Cl^−^ (6)

**Figure 10:**
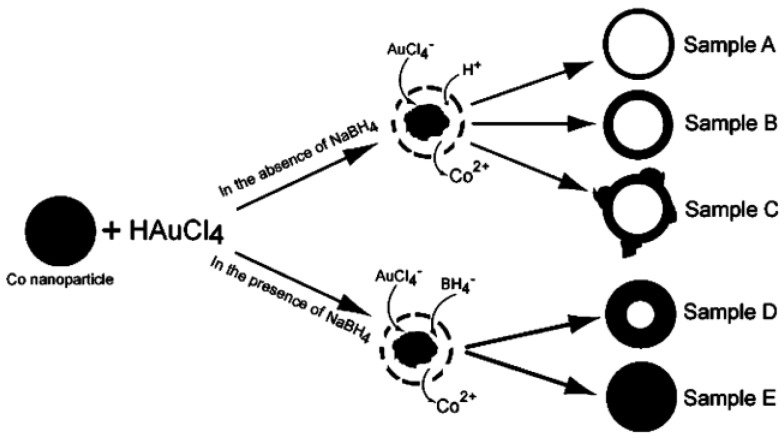
Synthesis of hollow gold nanoshells; role of NaBH_4_ in shell build-up. Reprinted with permission from Reference [64]. Copyright (2005) American Chemical Society.

Cobalt nanospheres not only act as template for gold nanoshell synthesis, but also as the reducing reagent. This is the reason that it is essential to remove excess NaBH_4_ before mixing HAuCl_4_ solution with cobalt nanospheres. If there is any NaBH_4_ remaining in the solution, thicker gold nanoshells are formed, which cause the distribution of shell thickness/core diameter to be wider (Figure 10).

**Figure 11:**
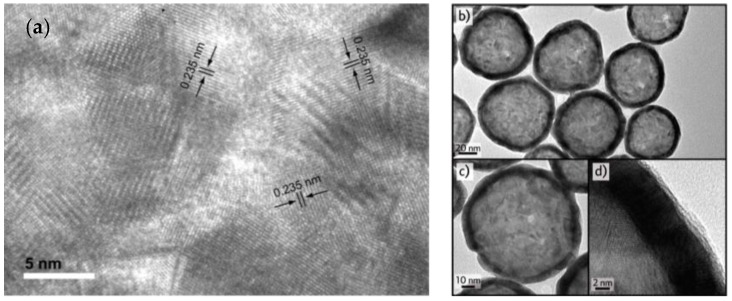
High resolution TEM images: (**a**) Depicting crystalline structure of gold nanoshells. Reprinted with permission from Reference [64]. Copyright (2005) American Chemical Society; (**b**–**d**) at various magnifications that detail hollow core (light) and shell structure (dark) Reprinted with permission from Reference [70]. Copyright (2014) American Chemical Society.

In addition, cobalt nanoparticle diameter determines the overall gold nanostructure, as gold atoms are confined to the vicinity of sacrificial template outer surface. As a result, synthesis of cobalt nanoparticles is the critical step of the whole process. After gold nanoshells are synthesized, the reaction flask is opened to air to oxidize the remaining cobalt, leading to the formation of a hollow core. Figure 11 shows high-resolution transmission electron microscopy (TEM) images of gold nanoshells. It was found that porous shells turned into dense surface after cobalt nanospheres were completely oxidized. This may be because of effective reconstruction of gold to form a smooth and highly crystalline structure (Figure 11a) through the Ostwald ripening process and with lower surface energy [64]. High resolution images at various magnifications (Figure 11b–d) clearly demonstrated build-up of a well-defined hollow core, and darker shell structure in these gold nanostructures [70].

##### (i) Effect of Growth Parameters on Hollow Gold Nanoshells Surface Plasmon Resonance Properties

**Figure 12:**
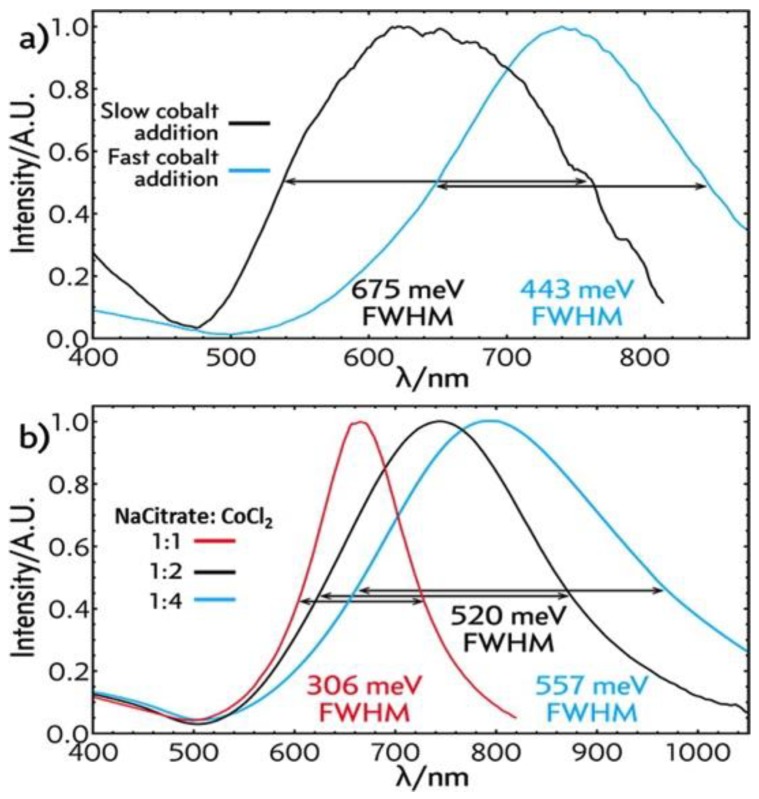
Monitoring gold nanoshell synthesis using UV-Vis absorption spectra: Mixing HAuCl_4_ solution with Co nanoparticles with slow addition (**a**) (black curve) and fast addition (blue curve); and effect of varying rations of CoCl_2_ and sodium citrate (**b**) (1:1, red; 1:2, black; 1:4, blue). Reprinted with permission from Reference [70]. Copyright (2014) American Chemical Society.

Schwartzberg group studied synthesis and growth mechanism of hollow gold nanoshells in detail, to understand factors that impact reproducibility [70]. They compared the effect of (i) fast and slow addition of cobalt nanospheres; (ii) CoCl_2_ to sodium citrate ratio; (iii) with and without PVP on cobalt nanospheres surface, and (iv) the effect of hydrolysis and growth time, on surface plasmon resonance properties of the resulting gold nanoshells. They used a cannula for slow dropwise addition, and for fast addition, cobalt nanospheres solution was poured directly into the container containing HAuCl_4_ solution. The results are summarized in Figure 12. For slow addition, not only the full width at half-maximum (FWHM) is broader than fast addition, but also there is a blue shift. It is because the reaction of the cobalt nanospheres with gold ion is extremely fast, and thus to get narrower FWHM, the two solutions have to be mixed quickly. Whereas the slow addition created higher concentration in a small area where cobalt nanoparticles solution dropped. Before it distributed to the entire gold ion solution, the cobalt nanoparticles were consumed by nearby gold ions. As a result, it created broader gold nanoshells diameters and thicknesses, as well as wider SPR, which is controlled by gold nanoshells diameter and wall thickness ratio.

**Figure 13:**
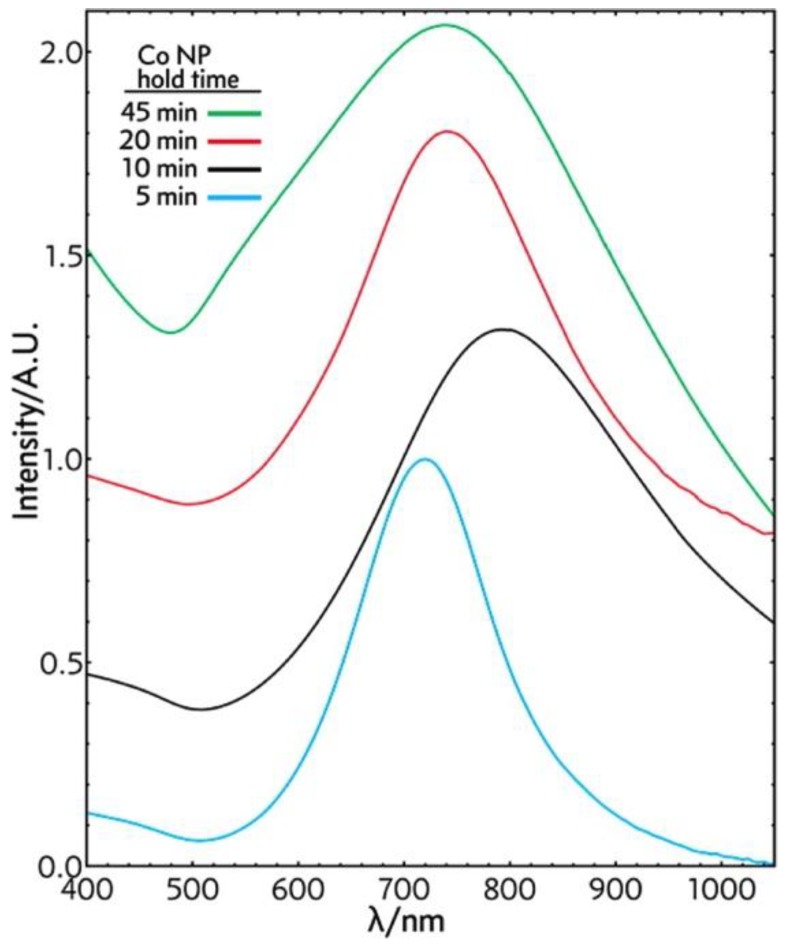
UV-Vis absorption spectral study of hollow gold nanoshell solution taken after NaBH_4_ addition—45 min (green), 20 min (red), 10 min (black), and 5 min (blue). Reprinted with permission from Reference [70]. Copyright (2014) American Chemical Society.

Schwartzberg group also examined the time it took for hydrolysis with sodium borohydride to be completed. As mentioned earlier, the presence of sodium borohydride affects the structure of gold nanoshells: By decreasing its concentration and increasing the concentration of sodium citrate, hydrolysis time is reduced from 45 min to 5 min. The corresponding SPRs are 730 (−143 meV), 800 (−266 meV), 760 (−186 meV) and 760 nm (−338 meV) for reaction times 5, 10, 20, 40 min respectively (Figure 13).

#### 2.2.2. Template Galvanic Replacement of Silver Method

Silver nanoparticles have also been used extensively as a template for the synthesis of hollow gold nanoshell [71]. The redox reaction between Ag^(0)^ and Au^3+^ is shown in the equation below:

3Ag + AuCl_4_^−^ → 3Ag^+^ + Au^0^ + 4Cl^−^ (7)

**Figure 14:**
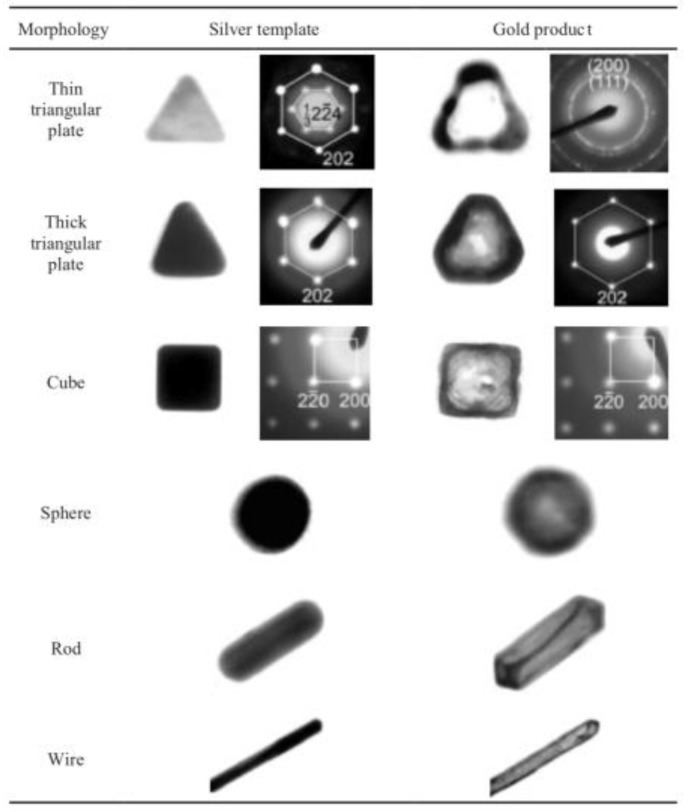
Silver nanoparticle morphology based gold shell synthesis. Reprinted with permission from Reference [72]. Copyright (2003) John Wiley and Sons.

Xia’s group first introduced this method of silver template replacement [72,73], in which they exploited a wide range of morphologies of silver nanoparticles to synthesize gold nanoshells (Figure 14). In their study, they demonstrated that the morphologies of gold nanoshells are determined by silver nanoparticle templates.

Synthesis of gold nanoshells by the silver template methodology has also been carried out in non-aqueous solvents such chloroform [74] and toluene [62]. Octadecylamine (ODA) was used to transfer freshly prepared silver nanoparticles into chloroform, and then mixed with chloroaurate. Using an organic solvent for the synthesis facilitates further surface modification of gold nanoshells.

**Figure 15:**
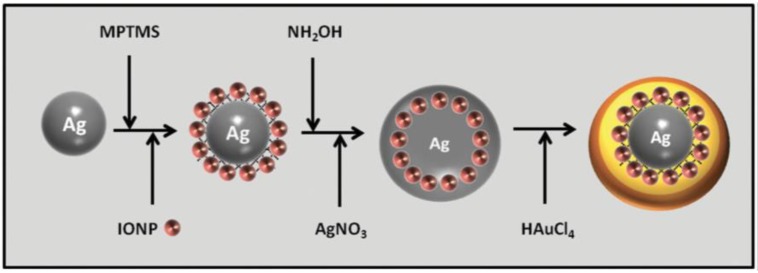
Synthesis of magnetic hollow gold nanoshells. Reprinted with permission from Reference [71]. Copyright (2014) John Wiley and Sons.

Drezek’s group exploited silver template method and encapsulated Fe_3_O_4_ nanoparticles inside hollow gold nanoshells, by decorating the silver core with Fe_3_O_4_ nanoparticles and growing another layer of silver as sacrificial template [71]. The synthetic scheme and final nanostructures are shown in Figure 15 [71].

#### 2.2.3. Electrochemical Synthesis of Hollow Gold Nanoshells

Methodologies described above for the construction of gold nanoshells require synthesis of cores as templates first, and the use of stabilizing and capping agents. This is followed by reduction of gold on the nanoparticle core templates [75]. It is important to control the chemistry of core build-up and the environment as the reaction of gold reduction is being carrying out. It has been demonstrated that small changes in these parameters can cause aggregation, and thus a loss of desired control of shell thickness. The need to have stabilizing and capping agents on gold nanoshells further hinders their surface modification for desired applications. A method to make “clean” hollow gold nanoshells on indium tin oxide (ITO) glass, was recently described, in which silver nanoparticles were electrodeposited on ITO surface without any reagents, organic ligands or surfactants as sacrificial templates [76]. Hollow gold nanoshells were then developed via galvanic replacement reaction between silver nanoparticles and HAuCl_4_ solution. Hollow gold nanoparticles synthesized by this method could be easily further functionalized with small organic molecules or macro-biomolecules [77,78,79].

#### 2.2.4. Nonmetallic Structures for Hollow Gold Nanoshell Synthesis

In addition to hard metals, soft templates, such as emulsion droplets [80,81,82] and microemulsions [83,84], have also been used to synthesize nanoscale hollow particles. There are several advantages of non-metallic structures core, as metal templates limit biological applications, and it is easier to incorporate guest species inside the soft templates.

##### (i) Emulsion Droplets

**Figure 16:**
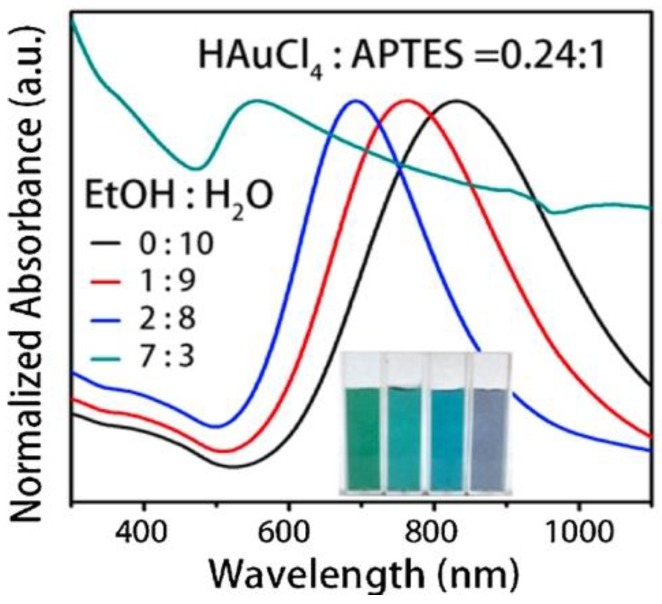
UV-Vis absorption spectra investigation of gold nanoshells synthesized through different ratios of ethanol/water mixture. Reprinted with permission from Reference [85]. Copyright (2016) Elsevier.

Surfactant assisted method is one way to synthesize hollow gold nanoshells. For example, Guan et al. synthesized hollow gold nanoshells by reducing HAuCl_4_ directly in 3-aminopropyltriethoxydisilane (APTES)-in-water suspension [85], in which APTES acts as a template in water. The diameter of gold nanoshells synthesized by this method ranged between 37–96 nm, and the corresponding surface plasmon resonance was in the range of 657–957 nm, which is in the visible and near infrared region. In a typical synthetic procedure, 10 μL of APTES were mixed with ~5 mL of water under stirring for 10 s to form a transparent colorless suspension, and then 0.40 mL of HAuCl_4_ (25 mM) was rapidly added, which formed a yellow suspension. The HAuCl_4_-APTES mixture was stirred for 30 s before adding 0.40 mL NaBH_4_. After the color changed to emerald green, 0.4 mL bist(trimethylsilyl)acetamine (BSA, 0.1 mM) was added to stabilize the resulting gold nanoshells. It was found that the size of gold nanoshells was tunable by simply adjusting the ratio of HAuCl_4_ to APTES or the ratio of ethanol/water in the reaction system (Figure 16). Besides APTES, gelatin has also been used as the template [80], and resulting nanoshells have a diameter of 20–40 nm and the wall thickness between 2–6 nm. Polylactide template prepared by the water-oil-in-water double emulsion formed lager hollow gold nanoshells [86].

In addition to surfactant-assisted methodology, other approaches were taken by using fast mechanical stirring stabilized emulsion method, to design surfactant free systems [87]. A phase transfer agent, tetraoctylammonium bromide (TOAB) is used to complex Au^3+^ ions and drive them to the organic phase, toluene. Then NaBH_4(aq)_ is added to the solution at a high stirring rate. Nanoscale water droplets containing NaBH_4_ are formed in the organic phase, which sequentially reduce gold to Au^(0)^, and form hollow gold nanoshells capped with dodecanethiol at the interface of toluene/H_2_O. This approach offers opportunities to encapsulate fluorescent dye and other nanoparticles for multi-tasking [87].

##### (ii) Biomaterial Templates

Biomaterials possess properties that make them good templates for metal nanoparticles synthesis [88,89,90,91,92]. For example, in addition to known composition and site-specific heterogenous surface chemistry, viruses are monodispersive with accessible interior, and extensive chemical tailorability. In general, there is a layer of proteinaceous shell, capsid, around viruses’ genomic material. There are several amino acids on the surface of capsid, such as cystine, glutamic acid and aspartic acid, that can act as anchor for precursor of gold nanoparticles. Besides, additional thiol groups can be easily added to viruses’ surface [88,89,93,94,95]. Thus, viruses are promising templates for gold nanoparticles. Utilizing viruses, metal nanoparticles with varied shapes can be synthesized, and a hollow interior can be easily constructed by removing core genetic material. In a typical procedure, virus stock solution without any surface modification is added directly to gold nanoparticles to attach the latter as seeds on the virus surface. Then a layer of gold is formed on the surface by reducing HAuCl_4_ with hydroxylamine hydrochloride [96].

## 3. Other Types of Gold Nanoshells

### 3.1. Multilayer Gold Nanoshells

Multilayer gold nanoshells show interesting surface plasmon resonance properties with multiple bands, which can be explained by plasmon resonance hybridization [97]. Multilayer gold nanoshells were first synthesized as insert gold nanospheres inside a gold nanoshell, with silica layer as a spacer. In these nanostructures, the surface plasmon resonance can be controlled not only by the diameter ratio of the spacer and gold nanoshells, and the gold nanoshells thickness, but also by the shape of the gold nanoparticle core and symmetry. Not only it is possible to manipulate surface plasmon resonance by these, but the absorption magnitudes and scattering cross section also rely strongly on these factors [98]. In addition, the outer layer gold nanoshells can act as optical condensers and focus light on the surface of the inner gold nanoparticle core, which can cause a significant near-field enhancement [99]. The effect of light focusing can be exploited in surface-enhanced Raman spectroscopy (SERS) applications, such as clinical diagnostics [100,101], environmental sensing, and biomedical research. Besides, fluorescence enhancement can also be achieved by tailored fluorophores in between inner core and outer gold nanoshells layer [102].

#### Synthesis of Multilayer Gold Nanoshells

Synthesis of silica spacer layer in the construction of multi-layer gold nanoshells was achieved by adopting the method of encapsulated gold nanoparticles with silica layer [103], and Stöber method for silica layer growth [104]. As a first step, APTES is added to gold nanoparticles solution, which not only provides amines as anchor sites, but also facilitates preparation of a thin silica layer (4–10 nm) by sequential condensation reaction with sodium [97]. For the synthesis of smaller gold nanoparticle core, lower concentrations of TEOS and NH_4_OH are used to grow silica layer to avoid charge destabilization at high pH and excess silane coupling agent [98]. Surface is then modified with APTES to provide amine groups for building a gold colloid layer as the precursor, before further reduction of HAuCl_4_ to form a complete gold layer [105]. The precise thickness of silica spacer can be controlled by over-growth using Stöber method, and etching silica layer by hydrolysis [102].

A different type of silica spacer was prepared by hydrolyzing 3-mercaptopropyltriethoxysilane (MPTES) in an alkaline medium. By coating the core with MPTES, a large number of thiols group are introduced onto the nanoparticle surface, which can act as an anchor for gold. It was followed by an in situ HAuCl_4_ reduction, leading to the formation of gold nanoshells in one-pot, and without the need to use gold colloid precursor [106]. Using MPTES avoids surface modifications with APTES to introduce NH_2_ groups for gold colloid precursor anchor, and the whole synthesis is carried out in an aqueous phase, which eliminates the process of phase transfer and repeated washing. Besides silica spacer, empty spacer can be built by coating a layer of silver on gold nanoparticle core as sacrificial template [107].

### 3.2. Anisotropic and Spiky Gold Nanoshells

Anisotropic spiky nanoparticles, such as gold nanostars [108,109] and nanoflowers [110], can generate intense electromagnetic field at their tips, which is similar to the lightning rod effect [111]. The strong localized fields at the tips enable this nanostructure with great potential for surface-enhanced Raman spectroscopy (SERS) [112,113], surface plasmon resonance platform for (bio)molecule detection [114], and/or nonlinear optical applications [115,116]. Compared to others, spiky nanoparticles have larger extinction cross section in the near-infrared (NIR) region, and as a consequence, photothermal heating capacity is greater than non-spiky nanoparticles, and it makes them a good candidate for diagnostic and therapeutic applications. The localized surface plasmon resonance can also be tuned by controlling the length, density and aspect ratio of the surface spikes. The surface resonance of spiky nanoparticles mainly comes from the plasmon mode hybridization of core and tips, especially for the spiky nanoparticles with short tips. By tuning the length of tips, the plasmon resonance can vary from visible to near-infrared. In addition, the core of spiky nanoparticles can act as an antenna to harvest light energy faster, and increase overall plasmon excitation cross section.

#### Synthesis of Anisotropic and Spiky Gold Nanoshells

**Figure 17:**
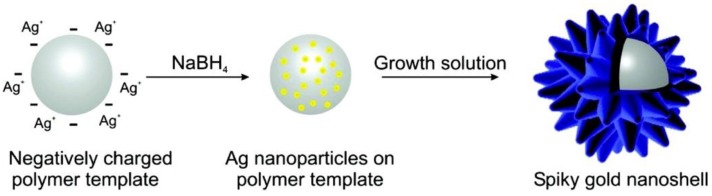
Spiky gold nanoshells synthesis. Reprinted with permission from Reference [118]. Copyright (2012) American Chemical Society.

Spiky gold nanoshells have been synthesized using a seed-mediated growth and structure-directing agents on self-assembled nanostructures of block copolymers or commercially available polymer beads [117,118,119]. For example, using self-assembled structures from block copolymers, iron oxide and ZnS-coated CdSe nanoparticles were incorporated onto the soft nanoparticles before growing spiky gold nanoshells on the core [117]. In a typical procedure, NaBH_4_ was used to reduce Ag(NH_3_)^2+^ to Ag nanoparticles (2–3 nm) as seeds on negatively charged core surface, and aged overnight to ensure that NaBH_4_ was fully consumed. Spiky gold nanoshells were grown by mixing seed-decorated cores with a growth solution containing partially reduced gold ions (Au^+^). The latter solution was prepared by mixing CATB, HAuCl_4_, AgNO_3_ and ascorbic acid at a molar ratio of 1:1.5:0.15:200 (Figure 17) [118]. The presence of silver ions in growth solution is important to control gold reduction rate and the shape of the nanoparticles.

**Figure 18:**
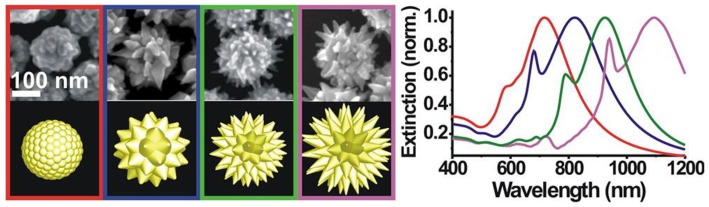
Spiky gold nanoshells: TEM images, morphologies and the corresponding extinction spectra. Reprinted with permission from Reference [19]. Copyright (2013) American Chemical Society.

Varied amounts and different types of surfactants as structure directing agents and ionic additives, have been used to control the topography from smooth shells to highly structured nanoshells, composed of spherical nanoparticles or sharp spikes of varying aspect ratios in the growth solution (Figure 18) [119]. Different concentrations of ascorbic acid were also employed to control the size and shape of spiky gold nanoshells on magnetic core [120]. Among different types of spiky gold nanoshells that have been synthesized, the disordered spiky gold nanoshells have larger Raman scattering cross section compared to the ordered ones [121]. Ling’s group used high molecular weight polymer, PVP, as structure directing agent to increase the length of spikes from 50 to 130 nm [122], and found that different spike lengths exhibited different optical responses.

**Figure 19:**
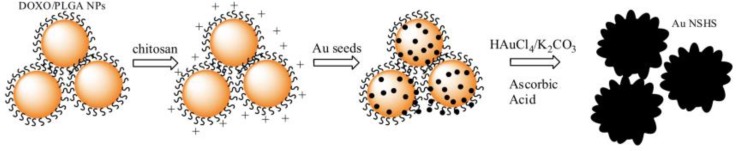
Schematics of synthesis of branched gold nanoshells. Reprinted with permission from Reference [123]. Copyright (2014) American Chemical Society.

Mosquera’s group took a different approach, in which seeded-growth surfactant-less method was used to synthesize branched gold nanoshells (BGNSHs) (Figure 19) [123]. They covered the core surface with chitosan that carried positive charge, and used it to bind to citrate capped gold seeds through electrostatic interaction. The growth solution contained HAuCl_4_ and K_2_CO_3_. In their design, the cores were loaded with chemotherapeutic drug, doxorubicin, and spiky gold nanoshells were surface functionalized with fluorescent near-infrared dye, protein human serum albumin, and folic acid. These moieties exert multifunctional abilities: Fluorescence imaging for diagnosis and therapy monitoring, combination chemotherapy, photodynamic and photothermal therapies. In addition to chitosan, glycol chitosan has also been used to synthesize such gold nanoshells [124]. In a similar approach, Lee’s group grew gold branches on iron oxide core with hydroquinone as reducing agent [125]. The main advantage of surfactant-less method is to have relatively clean and bare surface for easier further functionalization, whereas structure directing agents, such as cetyltrimethylammonium bromide (CTAB) and PVP, can strongly absorb on the surface, and thus hard to remove or modify.

**Figure 20:**
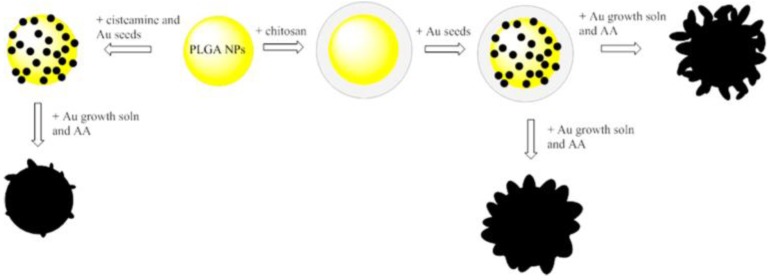
Control of anisotropy on branched gold nanoshells using chitosan or cysteamine as binding linkers for gold seeds. Reprinted with permission from Reference [126]. Copyright (2014) American Chemical Society.

A simple way to control different degrees of anisotropy and optical response was studied by tailoring the ratios of HAuCl_4_/K_2_CO_3_ (growth solution), ascorbic acid and NP-seed precursor. Chitosan and ascorbic acid (AA) are co-reductants and structure directing growth agents, which are important in determining the size and structure, as shown in Figure 20 [126].

## 4. Gold Nanoshell Stabilization

Nanoparticle solutions can be considered as colloidal suspensions, and one of the important issues with a colloidal solution is the stability of the dispersive system. This is because most colloids are thermodynamically unstable, and the dispersed particles tend to form larger particles, which eventually leads to aggregation. When this happens, the properties of nanoparticles change. In the case of nanoshells, it changes their plasmon resonance, and accordingly it is no longer determined by the ratio of wall thickness to diameter ratio of individual gold nanoshells, but by the secondary particles (aggregated gold nanoshells). This problem may occur during synthesis, storage, and even while attempting to study their applications. It clearly shows that stability is an important issue in gold nanoparticles. There are several forces in a colloidal system that need to be considered in order to stabilize it, and these include van der Waals, electrostatic, steric and solvation. Among these, electrostatic and steric forces are two that could be manipulated by modifying the surface of nanoparticles in order to stabilize them.

### Gold Nanoshell Electrostatic and Steric Stabilization

Citrate capped gold nanoparticles are an example of electrostatic stabilization, and this can generally be achieved by decorating the surface of nanoparticles with anionic or cationic ligands (such as surfactants, CTAB), and adjusting the pH of the solution to be below or above the isoelectric point. Another approach to stabilize nanomaterials is to decorate the surface with bulky ligands, such as polymers [127] or dendrimers [128]. There are few advantages of steric stabilization over electrostatic which include (i) less sensitivity to changes in ionic strength and pH; (ii) equal efficacy in both aqueous and non-aqueous solutions; and (iii) reversibility of flocculation.

#### Gold Nanoshell Stabilization: Surface Modification and Bioconjugation

Since the properties of gold nanoshells are strongly affected by their stability, it is important to consider surface modification of gold nanoshells, especially when it comes to biological applications. By using appropriate surface modification ligands, one can not only stabilize gold nanoshells, but also enhance their bioavailability. Polyethylene glycol is one of the most commonly used ligands for immune response suppression, and to minimize reticuloendothelial system clearance in the body [129]. PEGylated gold nanoshells have been shown to have distribution and elimination half-lives of 1.38 ± 0.38 and 71.82 ± 30.46 h in mice [18]. Antibodies, aptamers or peptide fragments have also been used for surface modification and bio-targeting purposes, and folic acid [130] and meso-2,3-dimercaptosuccinic acid (DMSA) [131] to increase cellular intake.

## 5. Characterization of Gold Nanoshells

The optical properties of gold nanoshells can be fine-tuned by choosing appropriate synthetic strategies, and adjusting the reaction conditions carefully. However, it is equally important to understand their physicochemical characteristics, as these strongly influence their physiological behavior. There are no standardized methods that are available to characterize metal nanoparticles in general and gold nanoshells in particular, in terms of their size, shell thickness, morphology and overall stability. A brief discussion of a combination of a variety of techniques that are used to characterize gold nanoshells, is included below.

### 5.1. Imaging Techniques: Transmission Electron Microscopy (TEM) and Scanning Electron Microscopy

The insight into composition and morphology of gold nanoshells can be gained by imaging techniques, including for example transmission electron microscopy (TEM) and high-resolution transmission electron microscopy (HRTEM). TEM relates to passing a focused beam of electrons through a sample deposited on a carbon grid [85], and using the transmitted beam to obtain information about the nanoparticles, at a spatial resolution as small as the atomic level dimensions [132]. The resolution of TEM varies considerably with the amount of material that electrons pass through, and the type of material (the heavier elements scatter more strongly and cause shorter free electron paths). TEM and HRTEM can also provide information related to the crystal structure and grain size [133]. The data from these imaging techniques can be combined with other analytical tools, such as electron energy loss spectrometry (EELS) or energy dispersive X-ray spectrometry (EDX), to provide additional information on the electronic structure and elemental composition of nanomaterials [133].

Scanning electron microscopy (SEM) is another imaging technique that employs electrons, and the difference between SEM and TEM being that the former uses electron scattering, whereas TEM images are generated by electron transmission. As a result, the SEM signals generated by scattering electrons reflect surface atomic composition and topographic details [134,135,136,137]. SEM and TEM provide similar information about nanoparticles i.e., size, crystallinity and lattice structure, however, SEM is more suitable for surface characterization.

### 5.2. Dynamic Light Scattering (DLS)

**Figure 21:**
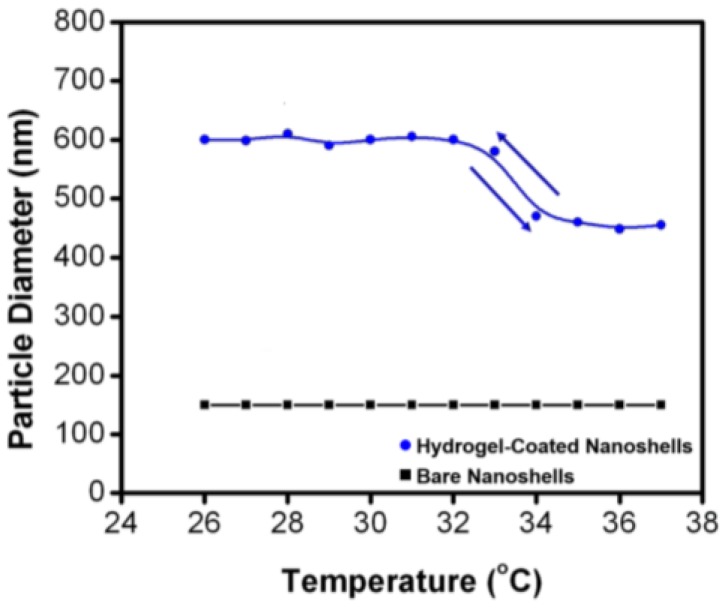
Variation of the hydrodynamic diameter of bare and hydrogel-coated gold nanoshells as a function of temperature, determined using Dynamic Light Scattering (DLS) [142].

DLS, also known as photon correlation spectroscopy (PCS), is one of the most common techniques to evaluate morphology and surface modification of gold nanoshells. DLS measures the fluctuations in the intensity of laser light that is scattered by the particles in Brownian motion in a sample solution, and determines the particle diffusion coefficient [138]. Hydrodynamic radii are calculated from the particle diffusion coefficient via Stoke-Einstein relationship [139]. As a result, a batch of similar gold nanoshells with different surface ligands may have different hydrodynamic radii [140]. It is a convenient and direct tool to measure surface modification by ligands, stability and aggregation of nanoparticles [141]. Besides, DLS studies can be carried out at controlled temperatures, which makes it suitable to study temperature based surface modification. As shown in Figure 21, bare gold nanoshells showed no change in hydrodynamic diameter in a temperature range, while it varied for hydrogel coated gold nanoshells based on temperature [142]. One of the limitations of DLS is that it assumes the particles to be spheres, which makes it impractical for size determination of nonspherical nanomaterials [143].

### 5.3. Zeta Potential (ς)

Zeta potential is another technique to analyze gold nanoshells. There are two layers of ions bound to nanoparticles: (i) The inner layer (Stern layer) which carries counter ions to surface charges, and can originate from surface ligand dissociation, or ions adsorbed on the nanoparticle surface; and (ii) the outer layer, a diffused layer which consists of loosely associated ions surrounding the inner layer [144]. The electrical potential at the boundary of this electrical double layer is known as zeta potential, and it can provide a good measure of the nanoparticle stability. As shown in Table 1, in general, nanoparticles with zeta potential over ±25 mV are considered stable colloidal systems [145]. However, one should use caution while expanding the scope of the principle of higher zeta potential to imply more colloidal stability, as steric stabilization may become of more relevance in metal nanoparticles. Surface functionalization of citrate stabilized gold nanoshells with linear and branched ligands via ligand displacement reactions, may lead to lower zeta potential, but more colloidally stable nanostructures, due to steric protection provided by these bulky ligands.

### 5.4. Energy-Dispersive X-ray Spectroscopy (EDX), X-ray Diffraction (XRD) and X-ray Photoelectron Spectroscopy (XPS)

EDX, XRD and XPS are common techniques for determining composition of nanomaterials. EDX spectrum is obtained by bombarding a sample with focused beam of electrons, and it provides elemental composition in bulk (μm), with limited information about speciation or oxidation states [146,147]. It has been used to confirm layer-by-layer synthesis of gold nanoshells starting from liposome to liposome-silica-gold nanoshells [148]. Similarly, using the sacrificial cobalt template synthesis approach, EDX was used to demonstrate full consumption of cobalt through oxidation of the template, and no cobalt was detected in the hollow gold nanoshells [149].

**Figure 22:**
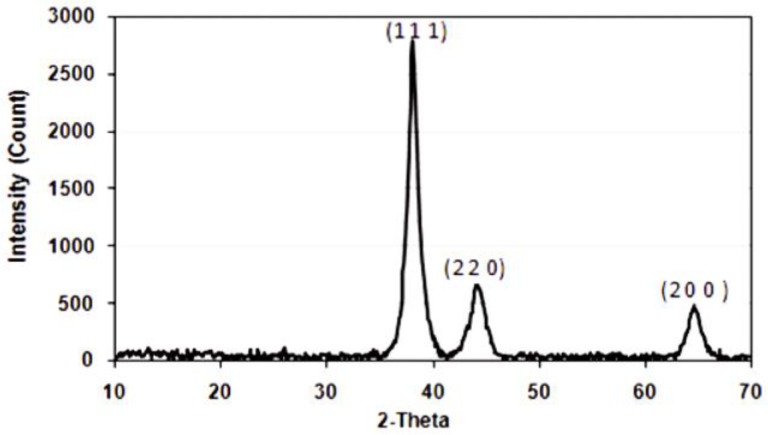
Face-centered-cubic (fcc) crystal lattice of silica cored gold nanoshells determined from their XRD spectrum [151].

XRD is another method to determine not only the composition, but also the structure (phase), and to calculate the size of nanoparticles by using Bragg’s law [150]. XRD spectrum from SiO_2_/gold core/shell nanoshells is shown in Figure 22, and according to which the crystalline lattice of silica core gold nanoshells was determined to be fcc [151].

**Figure 23:**
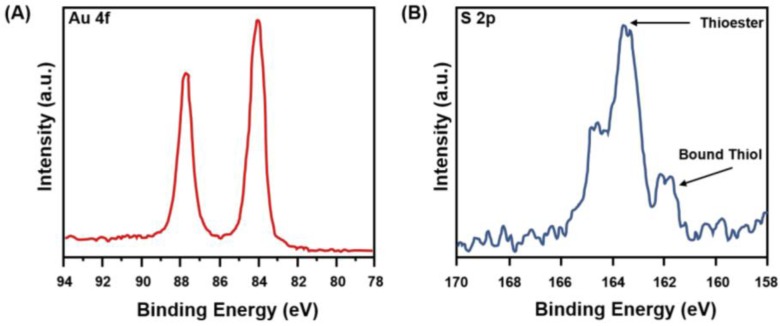
Au 4f (**A**) and S 2p (**B**) regions of the XPS spectra of HS-PEG2000-functionalized gold nanoshells [142].

XPS is commonly used for surface chemical analysis, and it is based on the principle of photoelectric effect, in which a sample is irradiated with X-rays, which excites electrons in specific bound states. XPS offers information related to elemental composition, electronic structure and oxidation states of elements. In addition, it can be used to monitor ligand-gold nanoshell chemistry [142]. As shown in Figure 23, the Au-S covalent bond formation was confirmed by XPS in the thiol-PEG functionalized gold nanoshells [142]. The binding energy for 2p_3/2_ electron in a free thiol is ~164 eV, while for the bound thiol on to gold, it is 162 eV [152].

### 5.5. Thermogravimetric Analysis (TGA)

TGA is a common technique to evaluate surface coverage of nanoparticles or the mass ratio of surface stabilizers to metal nanoparticles, by measuring mass loss with time over a temperature range. Upon heating a sample, components of a nanoparticle decompose at different temperatures, vaporize and cause a mass change. By plotting the weight loss against temperature, the surface composition of the metal nanoparticles can be calculated. It can also help measure surface density of ligands on gold nanoshells [149,153]. For example, generation 0 to generation 2 dendrimers were surface conjugated on gold nanoshells, and analyzed by TGA. It led to the determination of the dendrimer ligand density to be 63, 27, 7 ligands per nm^2^ for generation 0, 1, 2 dendrimers respectively [149].

### 5.6. UV-Vis Spectroscopy (UV-Vis)

**Figure 24:**
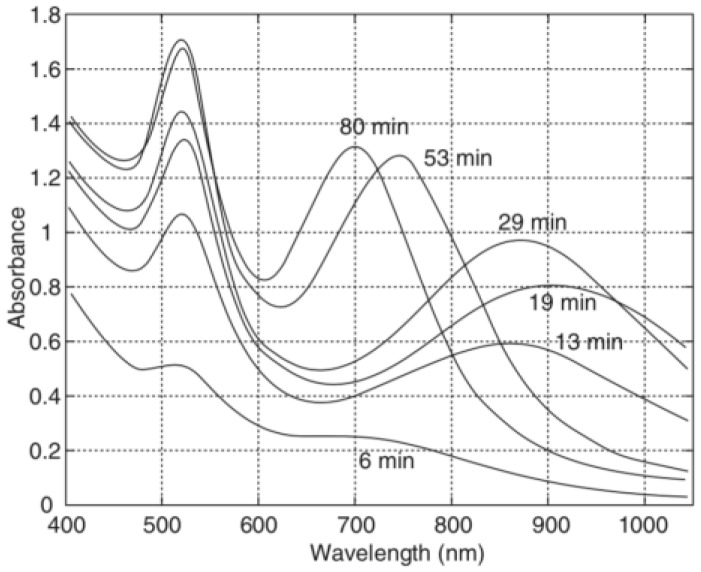
Monitoring gold nanoshell synthesis using changes in Ultra-violet/visible absorption spectrum. Reprinted with permission from Reference [155]. Copyright (2010) Wiley.

UV-Vis spectroscopy is an important tool to characterize metal nanoparticles, as their optical properties are sensitive to shape, size, concentration, aggregation, and refractive index near nanoparticle surface [154]. Since gold nanoshells are one of the plasmonic materials, UV-Vis spectroscopy is useful in so many different ways in characterizing these metal nanoparticles: (i) It has been used to monitor gold nanoshell synthesis: As shown in Figure 24, and complete nanoshell growth was confirmed by the appearance of characteristic plasmon extinction peaks [155]; (ii) to monitor surface coatings on gold nanoshell. It was found that the absorption of gold nanoshells changed upon coating with PVP, because of the increase in the dielectric constant of the medium [156]; and (iii) to monitor changes in shape of gold nanoshells. Drezek’s group used UV-Vis to demonstrate that using high energy laser for long period of time, hollow gold nanoshells can be restructured to gold nanospheres [71].

## 6. Conclusions

Gold nanoparticles and in particular gold nanoshells have intriguing physical properties, including optical and surface plasmon resonance, which can be tailored during their synthesis. When coupled with inherent biocompatibility and photothermal characteristics, gold nanoshells offer significant potential in nanomedicine, and are ideally suited for in vivo imaging studies. There has been tremendous effort devoted to chemical manipulation and fine-tuning of their overall nanostructure, to develop structure-property relationships that could then be used to suit a particular application in biology. Silica core based and hollow gold nanoshells are the most extensively studied nanoparticles, and synthetically each has met its own challenges. Build-up of the silica core and ligand stabilization of growing gold nanoparticles have been very well evaluated, and it is noted that the uniformity in gold nanoshell growth is significantly influenced by subtle changes in compositions of the reacting partners. Sacrificial galvanic replacement is the most commonly employed method for hollow gold nanoshell synthesis, but maintaining a batch-to-batch reproducibility seems to often fall prey to environmental conditions. Upon a careful analysis of all the synthetic effort in constructing core-based and hollow gold nanoshells, it is clear that there is still a lot of work needed to establish a correlation between reaction conditions and the desired morphology (size, shape, shell thickness) of gold nanoshells. There are challenges related to their reproducibility, which need to be addressed. Although tremendous progress has been made in developing characterization techniques for nanomaterials in general, there is a lack in expanding these into gold nanoshells, and obtaining a detailed understanding of their physical and physicochemical properties. Considering significant potential offered by gold nanoshells in resolving key issues in high morbidity rate diseases, including atherosclerosis, cancer, etc., it is believed that synthetic evolution will continue, and new methodologies will be developed that will simplify their construction and pave the way to clinical translation.

## Figures and Tables

**Table 1:** Stability behavior of colloids based on zeta potential values [145].

Zeta Potential Value (mV)	Stability Behavior
0 to ±5	Flocculation or coagulation
±10 to ±30	Incipient instability
±30 to ±40	Moderate stability
±40 to ±60	Good stability
Greater than ±60	Excellent stability
